# Editorial

**Published:** 1904-10-15

**Authors:** 


### EDITORIAL.

THE Editor of *Items of Interest* in the October number comments on the educational situation in a somewhat ambiguous fashion. In substance, he says it is no concern of the profession, or of the Examing Boards either, as to how many terms the colleges shall teach, or how long the terms shall be. In the same editorial he admits that there should be a minimum standard of length of term, and that a practitioner ought to have the right to say that his competitors "should have at least definite skill and attainments." He thinks a four-year high-school course should produce a better scholar than one of only three years. He however thinks a dental student who has had three years of training is ready to begin practice, and will have his business established by the time the four-year man is ready to commence his practice. From the position taken by the editorial we are unable to determine where the responsibility for this important decision should rest. The probabilities are that it never can be delegated to, or accepted from, any single course, since its influences are so far-reaching and important. Within the last two years we have had it brought very forcibly to our attention, that it does make considerable difference to people living on the other side of the globe, as to what kind of dental educational standards we maintain in the United States. American dentistry has secured a place of trustworthiness in the old countries that is a source of pride to every American who travels that way; but the American degree has received an almost irretrievable degradation there, because there was not sufficient authority in this country to compel a proper and decent educational standard. The truth of the matter is that it should be every one's business to use his influence to secure practicable ideal standards, whether he is directly or indirectly interested in teaching or practicing, or whether he is directly or indirectly interested in the quality of this or any other public professional service. The public good demands that no attainment in professional skill which ministers to the public health can be of too high an order. The old idea that professional healers are *called* to a sacred form of ministration is in great danger of being submerged under an avalanche of commercial greed. Why should we not turn about and look at our problem from the common sense and humanitarian point of view? We suppose however that this may never come to pass; not because we are so dead to the best interests of our profession and the welfare of our clients that we have lost human sympathy and interest, but because our social environment and business methods, and like obligations, are such that we haven't the moral courage to do those things which tend to the development of the best interests of all concerned. Like many other teachers, we have lost all hope that the dental colleges of this country, through their association or as individuals, will, in the near future, avow an educational standard that shall promise any considerable advance in attainment for their graduates. We therefore are very willing that the National Board of Examiners shall try its hand at establishing a standard which shall be the unbiased judgment of the profession at large, rather than the dictum of the colleges, which must be more or less self-interested. As we think of it, this organization should by rights perform this function. It represents in the first place the people for whose welfare all laws are supposed to be made, and secondly it represents the profession from which its members are all selected. It is under no obligation to favor the dental college as a business proposition, but as an educational institution it is in duty bound to increase its efficiency in every practicable way. It can not therefore demand the impossible from the college without jeopardizing the interests it is pledged to conserve. The fear expressed by some college men that the Examiners may become arrogant and offensively dictatorial can not materialize without peril to its own organization. Should this organization unite in its membership all the States of the Union and agree to recognize and license for practice, graduates having had a fixed and reasonable amount of mental and technical training, the colleges would quickly and willingly comply, and the problem of uniform interstate reciprocity and legislation would naturally follow, and without knowing it we should have a national standard of professional education. Where, then, would the fake diploma mills have a chance to discredit our diploma? Let's turn the job over to the National Board of Examiners, and do all we can to assist in establishing on a perpetually sound basis our educational standards. This will free the members of our faculties from the burdens of vexatious business cares, for the more delightful and profitable study and practice of pedagogy.

